# Automatic Radiographic Position Recognition from Image Frequency and Intensity

**DOI:** 10.1155/2017/2727686

**Published:** 2017-09-17

**Authors:** Ning-ning Ren, An-ran Ma, Li-bo Han, Yong Sun, Yan Shao, Jian-feng Qiu

**Affiliations:** ^1^College of Radiology, Taishan Medical University, Taian, Shandong, China; ^2^College of Information and Engineering, Taishan Medical University, Taian, Shandong, China; ^3^Department of Radiology, Affiliated Hospital of Taishan Medical University, Taian, Shandong, China

## Abstract

**Purpose:**

With the development of digital X-ray imaging and processing methods, the categorization and analysis of massive digital radiographic images need to be automatically finished. What is crucial in this processing is the automatic retrieval and recognition of radiographic position. To address these concerns, we developed an automatic method to identify a patient's position and body region using only frequency curve classification and gray matching.

**Methods:**

Our new method is combined with frequency analysis and gray image matching. The radiographic position was determined from frequency similarity and amplitude classification. The body region recognition was performed by image matching in the whole-body phantom image with prior knowledge of templates. The whole-body phantom image was stitched by radiological images of different parts.

**Results:**

The proposed method can automatically retrieve and recognize the radiographic position and body region using frequency and intensity information. It replaces 2D image retrieval with 1D frequency curve classification, with higher speed and accuracy up to 93.78%.

**Conclusion:**

The proposed method is able to outperform the digital X-ray image's position recognition with a limited time cost and a simple algorithm. The frequency information of radiography can make image classification quicker and more accurate.

## 1. Introduction

Digital X-ray imaging technique has generated massive amounts of clinical image data in radiology departments every day. These data need to be classified, retrieved, and analyzed in Picture Archiving and Communication Systems (PACS) or Radiology Information Systems (RIS). The urgent requirements to process these massive image data demand an automated and computationally efficient approach [1, 2]. Among these approaches, image classification, radiographic position identification, and artificial intelligence analysis are the most widely used ones. In this sense, the retrieval of images and the learning of radiographic position are the most fundamental parts.

Traditional medical image retrieval is semimanual, which obtains clinical information from manually retrieved image annotations and databases. The disadvantage of this approach involves human errors and operator variations, which is labor intensive and results in lower accuracy [1]. Automated methods using image retrieval technique are based on image features such as color [2, 3], texture [4], and shape [5]. Wang et al. proposed a dynamic interpolation method to achieve stereo microscopic measurements, but the scheme required a large quantity of matching elements [6]. Histograms were also widely used for image retrieval but had its relevant disadvantages [7, 8]. Other image retrieval techniques such as wavelet transform (WT) [9], Fourier transform (FT) [10], local binary pattern (LBP) [11], and Tamura texture features [12] can recognize an image type through library searching and image classification. However, position information cannot be automatically determined with these algorithms. Besides, these methods lack recognition on imaging organ tracking as researched by Jiao et al. [13].

Pattern recognition can automatically process and analyze digital images as mentioned by Paparo et al. [14, 15]. Feature selection method reported by Silva et al. [16] and Hussain [17] was used in traditional learning algorithms such as support vector machine (SVM) and k-means for image retrieval but needs large datasets for training. Medical expert systems as discussed elsewhere [18, 19] used mixed algorithm to extract target area. Multilayer perceptron neural networks (MLPNN) can identify tissues and diseases as discussed in other places [20–22]; however, the process is complex and the processing time is too long for clinical use. Recently, the well-known deep learning algorithm has also been introduced to medical image processing and achieved equivalent results compared with professional expertise [23–25], but the data quantity and accuracy have remained a debate [26].

Therefore, in this paper, a method that combines frequency curve classification with gray scale matching for image retrieval and matching is proposed. It uses a whole-body phantom image as the template mask for anatomical and radiographic location marking, with shorter time cost and higher accuracy.

## 2. Materials and Methods

### 2.1. Image Preprocessing

Raw digital radiographic image data typically has large dynamic range and gray level features. Therefore, we use linear histogram stretching and a median filter for noise reduction. The respective equations are

(1) $$\begin{array}{c} F_{H}\left(x,y\right)=\left(\frac{65535}{B-A}\right)\cdot \left[f\left(x,y\right)-A\right], \end{array}$$

where

(2) $$\begin{array}{c} A=\text{min}\left[f\left(x,y\right)\right],B=\text{max}\left[f\left(x,y\right)\right], \end{array}$$

and

(3) $$\begin{array}{c} F_{w}\left(x,y\right)=med\left\{f\left(x-R,y-l\right),\left(R,l\right)\in w\right\}. \end{array}$$

*w* is 5.

### 2.2. The Phantom X-Ray Image Masks

X-ray imaging phantoms are physical analogs of human body shapes and tissues as studied by Dewerd and Kissick [27]. Plastic and nylon are used to simulate the outline of the human body, bones, and primary tissues for whole-body radiography. We took X-ray imaging of the brain, cervical spine, chest, lumbar spine, pelvis, and limbs of a whole-body phantom (Whole Body Phantom PBU-50, Kyoto Kagaku, Japan) by using Digital Radiology DR (Wan dong HF50, Beijing, China). Each of the images was processed by adjusting the histogram, filtering, performing rigid translations, and scaling [28] and then fitted into a whole-body radiographic image. We also performed contrast-limited adaptive histogram equalization (CLAHE) for handling the variation in X-ray exposures.

**Figure 1:**
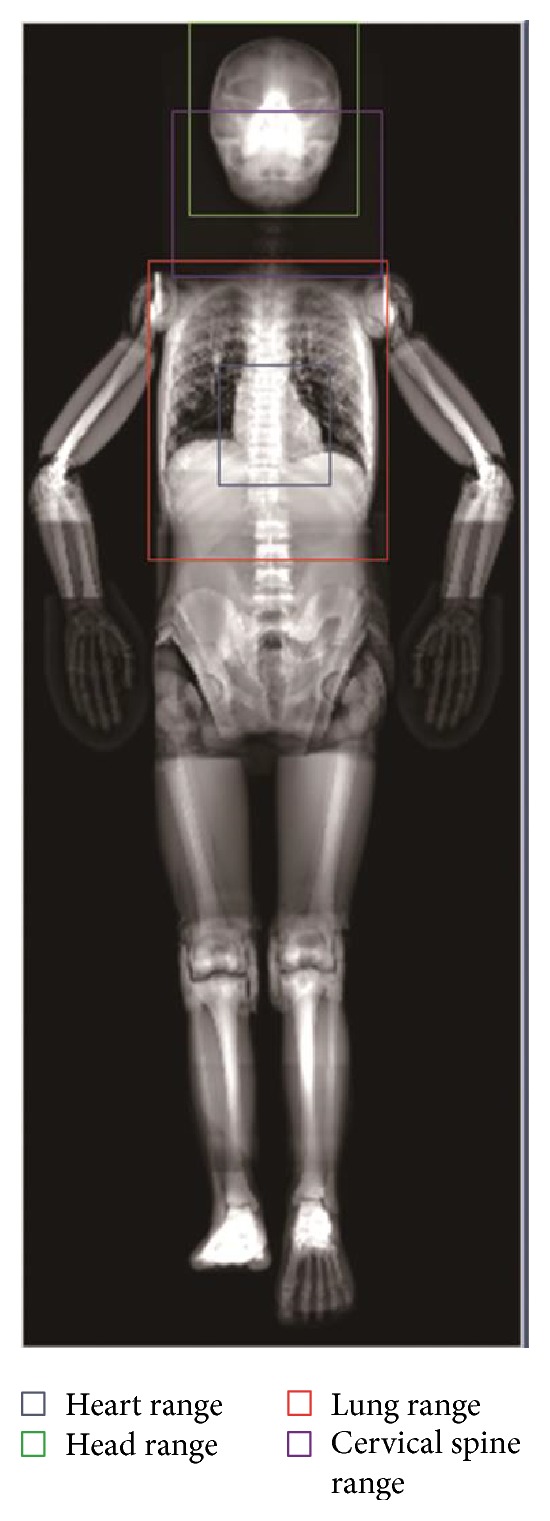
The whole-body phantom's X-ray mask and the examples of partial anatomical definition.

For recognition of the radiographic positions after completing the input image matching, we performed the anatomical definition to a phantom template; the matrix of images is 2000 × 800, and the height of the corresponding body is 165 cm without gender. For the information of the image, diagnostician can use different ranges to define different organs, such as head size ranging from [260, 1] to [540, 285] and lung size ranging from [250, 130] to [560, 365], as shown in Figure 1. For the frontal image, there are seven radiographic positions and six radiographic target organs. The phantom template defines the target template for subsequent matching based on automatic identification and X-ray photography posture.

### 2.3. Classification Based on Image Frequency

Radiographic images have special frequency and amplitude characteristics, which are position dependent. These characteristics of the frequency curve can be used for classifying the type of image (for a given radiological position) and extract the texture of the organ.

### 2.4. The Characteristics of X-Ray Image Frequency

**Figure 2:**
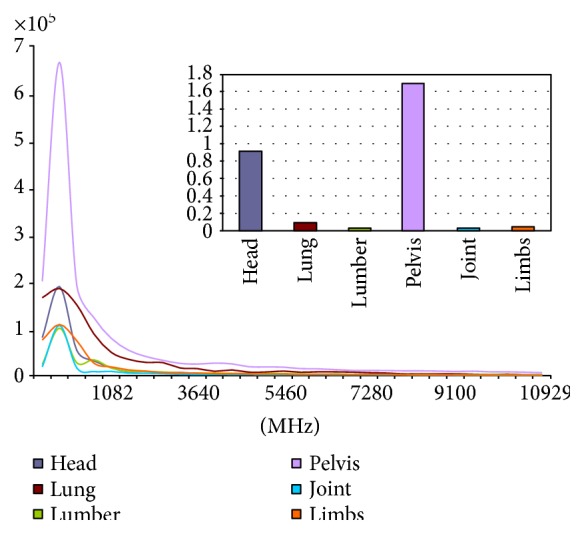
Frequency curves and the AUCs for various anatomical regions.

We use the fast Fourier transform (FFT) of the organ images to obtain the frequency spectrum as follows:

(4) $$\begin{array}{c} F\left(u,v\right)=\frac{1}{MN}\overset{M-1}{\underset{x=0}{\sum }}\overset{N-1}{\underset{y=0}{\sum }}f\left(x,y\right)\cdot e^{-j2\pi \left(ux/M+vy/N\right)}, \end{array}$$

where *M* and *N* are the image resolution and *u* and *v* are coordinates in the frequency domain. From the frequency image and 2D curve, we find that the effective anatomical contours concentrate on the minimum 2% of the frequency curve. In Figure 2, the frequency curve at each position is the average of 10 images of the same radiological position in the same coordinate system, and the curve features shown are significantly different among the positions. Partly, the differences of some positions such as the lungs and limbs are not reflected in the frequency curve; thus, we offer the areas under the curve (AUCs), whose values of the lungs and limbs have obvious differences. Combined with frequency curve and AUCs, differences of positions can be obviously shown. The radiological positions are the head, lungs, lumbar (spine), pelvis (abdominal), joint (knee), and limbs.

**Figure 3:**
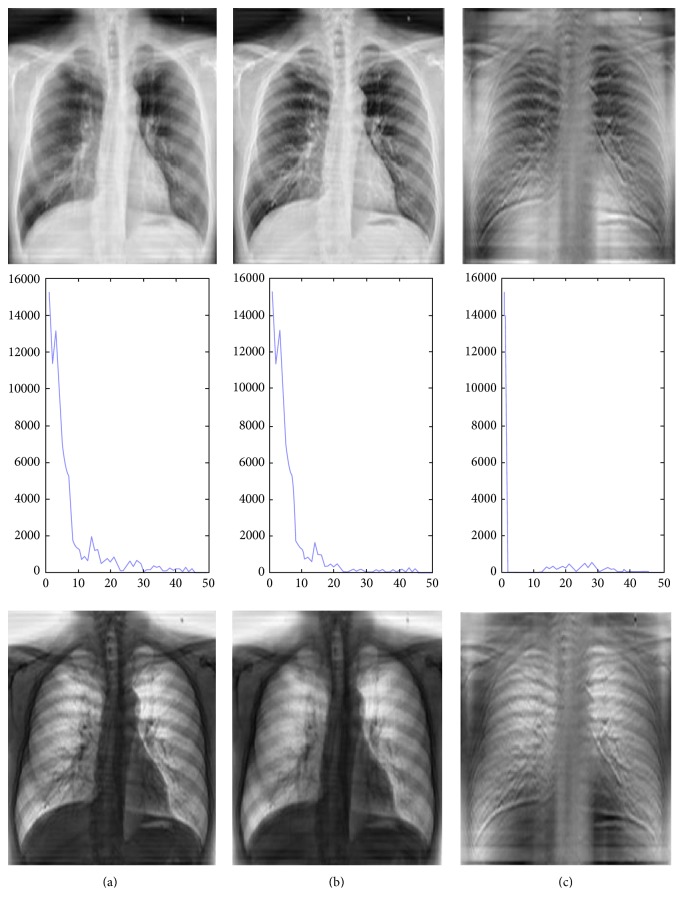
(a) From top to bottom: the chest X-ray image, the image frequency curve, and the chest X-ray image with inversed gray scale. (b) From top to bottom: chest X-ray image by Butterworth filtering, the image frequency curve, and the chest X-ray image with inversed gray scale. (c) From top to bottom: lung texture image reconstructed by the filtered frequency information, the frequency curve, and the lung texture image with inversed gray scale.

**Figure 4:**
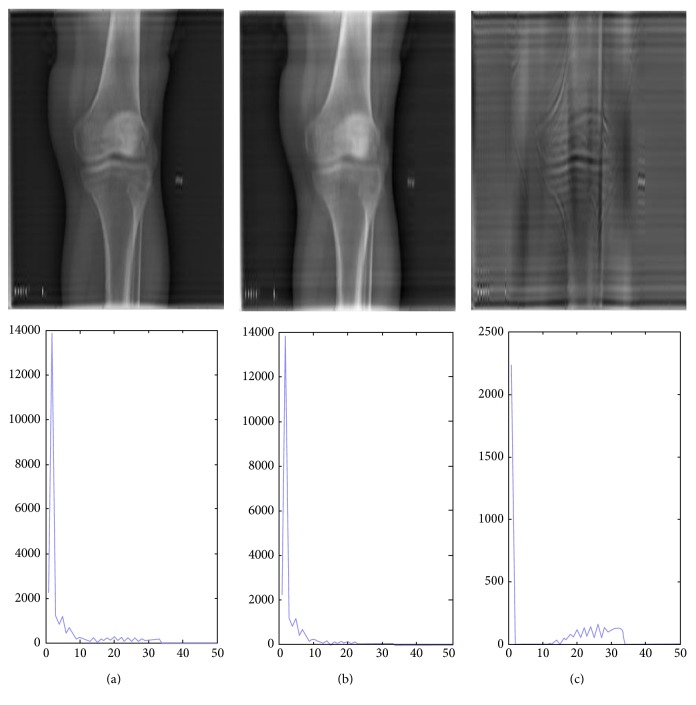
(a) From top to bottom: the knee X-ray image and the knee image frequency curve. (b) From top to bottom: the knee X-ray image by Butterworth filtering and the image frequency curve by filtering. (c) From top to bottom: the trabeculae texture image reconstructed by the filtered frequency information and the frequency curve.

In X-ray images, organs or tissue has a characteristic frequency response, even in different samples and different radiological positions. For example, the chest imaging using appropriate exposure parameters shows lung texture details and the lung signal captured in certain frequency bands. As shown in Figure 3, which shows the average frequency curve of 10 lung X-ray images, there is a peak in the low-frequency range, which corresponds to a lung texture detail (extracted using a Butterworth filter). For comparison, a similar peak in the averaged knee curve corresponds to bone trabeculae as plotted in Figure 4.

### 2.5. Classification Based on Image Frequency

The frequency curves for six radiographic positions were used as the standard library for comparison with arbitrary input images, and the similarity between input image and standard library was determined by the mean variance of the vector frequency curve. The input image is *f*(*w*); the corresponding amplitude-arranged vector is [*A*1, *A*2,…, *An*]; the six frequency curves, *F*(*w*), are used as a standard for comparison with arbitrary input images in the library and have amplitudes of [*B*1, *B*2,…, *Bn*]. The mean-variance similarity between the input image and the reference organ image is

(5) $$\begin{array}{c} a=sqrt\left(\frac{\sum_{i=1}^{n}\left(\left(f\left(w\right)-F\left(w\right)\right)\cdot^{\wedge }2\right)}{length\left(f\left(w\right)\right)}\right),\\ a=sqrt\left(\frac{sum\left(\left(f\left(w\right)-F\left(w\right)\right)\cdot^{\wedge }2\right)}{length\left(f\left(w\right)\right)}\right). \end{array}$$

The cosine of the angle *θ* between the two images can be described as follows:

(6) $$\begin{array}{c} \text{cos }\theta =\frac{\sum_{i=1}^{n}\left(f\left(w\right)\cdot F\left(w\right)\right)}{\sqrt{\sum_{i=1}^{n}{\left(f\left(w\right)\right)}^{2}}\cdot \sqrt{\sum_{i=1}^{n}{\left(F\left(w\right)\right)}^{2}}}=\frac{f\left(w\right)\cdot F\left(w\right)}{\left|f\left(w\right)\right|\cdot \left|F\left(w\right)\right|}. \end{array}$$

**Figure 5:**
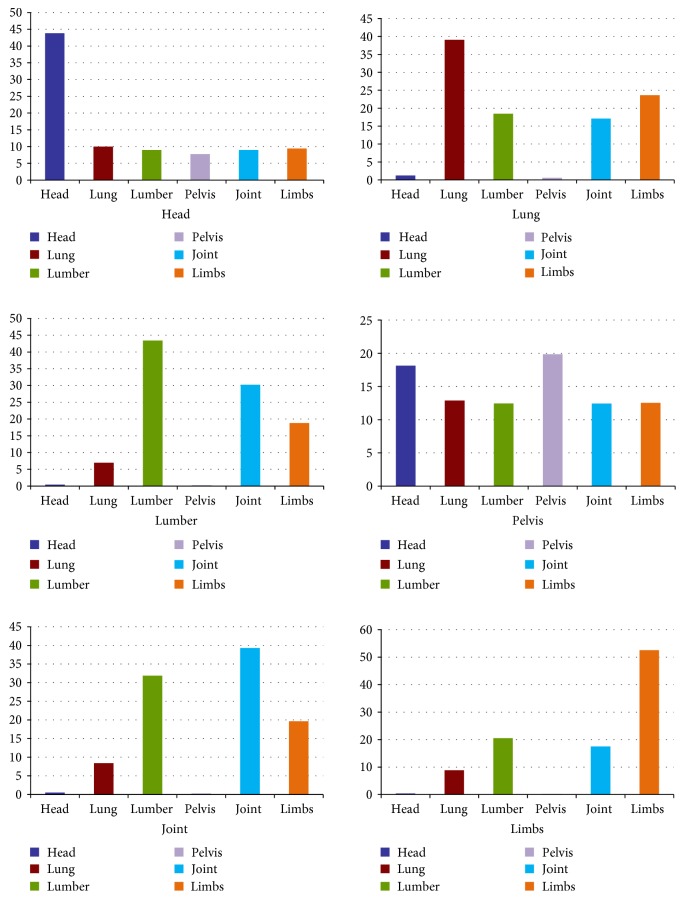
The reciprocal of mean-variance between 6 organs and the standard frequency curve.

The smaller mean-variance is and the closer the cosine value is to 1 (indicates an angle closer to zero), the greater the similarity is. Matching 6 curves yields 6 mean-variance values, and then bubble sort is performed to determine the two mean-variances with the highest absolute value. The absolute values of the top two are less than 0.02, comparing the cosine similarity between the wave curves of the source image and organs which corresponds to the top two mean-variances. The organ which is the closest to mean-variance is considered the same as the organ of the source image. Six organs had mean-variances with standard frequency curves, and the reciprocal of that for all organs is plotted in Figure 5(), as histograms. Higher reciprocal of mean-variance signifies greater similarity.

### 2.6. Image Matching Based on Matrix Multiplication and Correlation Coefficient

After the vector calculations based on image frequency have been performed, we determine the types of the images that are the most similar to the standard organ curve according to the shape of their curves and mean variances. The input image will be matched against the whole-body phantom mask so that the organ field is defined. This step involves matrix multiplication and the correlation coefficient.

In (7), (8), and (9), the input image after preprocessing is *F*_input_(*x*, *y*) and the 2% part of the frequency curve is *f*(*w*). The image has been finished by classification based on image frequency, and the phantom image is denoted as *p*(*m*, *n*). (In (7), (8), and (9), *p*(*m*, *n*) represents image patches whose frequency is not within the minimum 2% range. The range of *p*(*m*, *n*) represents the image from top to bottom.) By finding the maximum values of *M* and *R*, region *T* can be found, which is the intersection of *M* and *R* shown in the phantom image and also the target recognized region.

(7) $$\begin{array}{c} M=\frac{F_{input}\left(x,y\right)\cdot p\left(m,n\right)}{\sqrt{F_{input}{\left(x,y\right)}^{2}}}=\frac{\sum_{m}\sum_{n}F_{input}\left(x,y\right)\cdot p\left(m,n\right)}{\sqrt{\sum_{m}\sum_{n}{\left(F_{input}\left(x,y\right)\right)}^{2}}}, \end{array}$$

(8) $$\begin{array}{c} R=\frac{\sum_{m}\sum_{n}F_{input}\left(\left(x,y\right)-\bar{\sum_{m}\sum_{n}F_{input}\left(x,y\right)}\right)\cdot \left(p\left(m,n\right)-\bar{\sum_{m}\sum_{n}\;p\left(m,n\right)}\right)}{\sqrt{\sum_{m}\sum_{n}{\left(F_{input}\left(x,y\right)-\bar{\sum_{m}\sum_{n}F_{input}\left(x,y\right)}\right)}^{2}\cdot {\left(p\left(m,n\right)-\bar{\sum_{m}\sum_{n}\;p\left(m,n\right)}\right)}^{2}}}, \end{array}$$

(9) $$\begin{array}{c} T=M\left|R\right.. \end{array}$$

The maximum values of *M* and *R* have been solved, respectively, by the matrix multiplication and correlation coefficient, between the input image and phantom image. *T* is a region corresponding to the phantom area and is indicated by a bright box. To improve the processing speed, the matrix of the input and phantom images is reduced (maintaining image proportions).

### 2.7. Implementation of the Overall Algorithm

**Figure 6:**
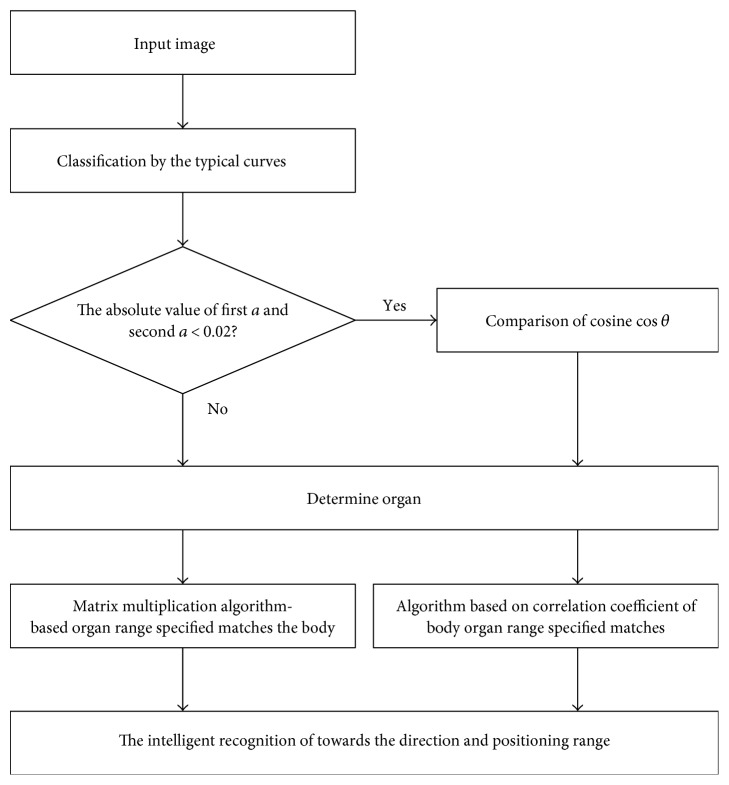
Workflow.

For any input image being preprocessed, the 2D Fourier transform will be taken and the lowest 2% frequency curve of the image is obtained. Compared with 6 predefined curve types and the input image type (radiographic position), the curve is classified by calculating the curve similarity and the mean variance. Next, the image is matched in the phantom image by finding the maximum value of matrix similarity. The final matching region, which corresponds to a priori knowledge of the patient's anatomical field, is shown in the phantom as the result. The workflow is shown in Figure 6.

## 3. Results and Discussion

217 clinical radiological images were randomly collected in this study, from the Radiology Department of Taishan Medical University. The radiological position and body region in all images have been automatically recognized by our method. The results were verified by the clinical physicians of the Radiology Department. For comparison, the input images were also processed by dot matrix matching, correlation matching, and histogram retrieval algorithms. The accuracy rates and the processing times are shown in Table 1. The accuracy between the proposed method and any other methods has a statistically significant difference (*p* < 0.005).

The results have shown that the proposed algorithm has the highest accuracy and robustness for all images (6 position types); the average organ recognition accuracy was 93.78% and the average judgment time was 0.2903 s.

The proposed method is better than other benchmark methods; moreover, the method can obtain the radiographic position's description from the anatomical knowledge in the phantom image and reduce the processing time and recognition accuracy. What is more, compared with some effective approach such as the large margin local estimate (LMLE) [15] and deep learning network [24], the LMLE method only achieved less than 90% accuracy with 10% data as the training set. Although the convolution neural network in [24] achieved more than 90% accuracy in most image data, the approach needs 7000+ image slices and a most recently equipped computer (i7 3.4GHz, 16 GB RAM) for neural network training, while our method only needs simple matrix multiplication and correlation coefficient which can be calculated on a multicore computer with less time and more than 90% of the accuracy.

**Figure 7:**
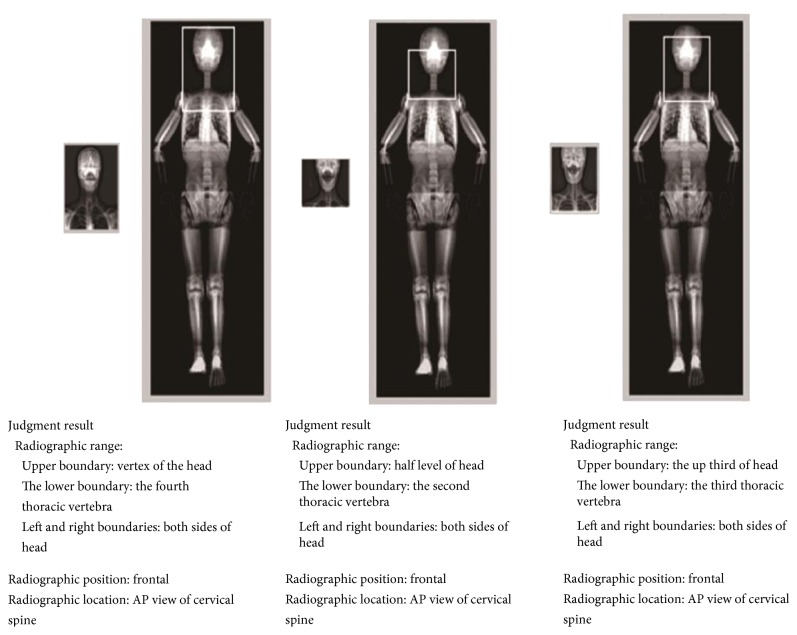
The automatic recognition results for three cervical spine images.

The sample results of the radiographic position recognition are shown in Figure 7, by matching rectangular areas and annotated text. This integrated method can accurately mark the photograph site on the phantom images. We can get the information of photography range and photography sites according to early anatomical definition in phantom-pixel area. For different images with the same position type, the image matching can show the regional differences in the whole-body phantom image. For example, in Figure 7, three different cervical spine images have been identified and shown in different cover areas.

The human body model was represented by a phantom template X-ray image. The phantom was developed to mimic the human body X-ray attenuation parameters. The radiography of the phantom was closely approximated to the real human, even though the model structure was only simplified to the macroscopic shape of the organs. For example, the lung phantom made of plastic can simulate the lung contour and segments but did not include the pulmonary veins and nodules. In the X-ray image of the phantom, the macroscopic profile of the lung is authentic for the imaging modality. The majority of conventional radiography sites are matched accurately by using this phantom image approach. For the detection of the contours of the lungs and the heart, the independent frequency or gray information is not sufficient.

The histogram and gray intensity are widely used for image similarity detection. Histogram matching has the advantage of being fast and no limitation by image size. However, it cannot determine the position and scope information. The method presented in this paper obtains robust frequency characteristic curves from X-ray information. The templates of different anatomical features have distinct frequencies and amplitudes. Comparison of input images and template only needs to take 2% effective frequency characteristics.

We extract a 1D curve from a 2D image, which accelerates and simplifies the image-matching algorithm. For 5.5 GB image data consisting of 217 images, the total processing time was 414.6 s.

Although our method was performed well for all of the test images, the algorithm has some limitations. The major obstacle is the poor result for nonstandard radiography; the matched result will be in the wrong position in the phantom image. For these cases, in a subsequent study, we plan to develop more standard phantom models, such as for babies, animals, and separate male and female bodies, in order to obtain more appropriate phantom images.

## 4. Conclusions

In this paper, we proposed a method for the automatic recognition of a radiographic position and body field, based on frequency curve classification and gray information of digital radiographic images. Compared with image analysis methods based on complex pattern recognition algorithm, the proposed method can extract more information about the patient's position. The frequency classification in this work has good sensitivity and robustness to reduce the errors, which is caused by variations in the lighting environment (image exposure, detector sensitivity). This method is a fast 1D classification for 2D images and can be used for automatic feature extraction and be applied to big data calculations.

## Figures and Tables

**Table 1:** The accuracy rate of four different radiographic position matching methods.

Radiographic positions	Dot matrix matching algorithm (%)	Correlation matching algorithm (%)	Histogram retrieval algorithm (%)	Current algorithm (%)	Average time of current algorithm (s)
Head	83.3	100.0	50.0	100.0	0.2808
Lungs	47.4	71.9	45.6	100.0	0.2918
Lumbar	45.6	66.7	40.5	100.0	0.2934
Pelvis	35.3	41.2	41.2	66.7	0.2919
Joint	90.9	100.0	27.3	100.0	0.2903
Limbs	75.8	56.8	56.6	96.0	0.2936
Average	63.1	72.7	43.5	93.7	0.2903
